# Do expectations shape interoceptive perceptions across body domains? A sham EMF study to test the predictive processing theory

**DOI:** 10.1016/j.ijchp.2025.100609

**Published:** 2025-07-18

**Authors:** Natalie Schmitz, Carolin Wolters, Antonia Rahrbach, Friederike Kälke, Michael Witthöft, Alexander L. Gerlach, Anna Pohl

**Affiliations:** a University of Cologne, Institute of Clinical Psychology and Psychotherapy, Pohligstr. 1, 50969 Cologne, Germany; b Johannes Gutenberg University Mainz, Institute of Clinical Psychology, Psychotherapy, and Experimental Psychopathology, Wallstr. 1, 55122 Mainz, Germany

**Keywords:** Predictive processing, Interoception, Somatic symptom burden, Response bias, Cardiovascular signal detection task, Somatic signal detection task

## Abstract

**Objective:**

According to the principles of predictive processing theory, persistent symptom perception is largely determined by central nervous predictions on somatosensory input. Here, we examine how threat-related expectations shape predictions and interoceptive perceptions across body domains using sham EMF (electromagnetic field) exposure.

**Methods:**

Participants (*n* = 113) were recruited via announcements at the university. Most participants were female (76.1 %) with a mean age of 25.12 years. Participants were divided into two groups (sham EMF on/off). Both groups completed a somatic and a cardiovascular signal detection task (SSDT, cvSDT) in pseudo-randomized order. Sensitivities and response biases were calculated. Self-reports (symptom distress, anxiety) were completed. Group effects were analysed with (M)AN(C)OVAs. In four exploratory regression models response bias and anxiety (state/trait) served as predictors for somatic symptom distress.

**Results:**

Participants in the sham EMF group reported significantly higher levels of state anxiety (*p* = .021, *d* = 0.44) and, trend-wise, more symptoms during the experiment (*p* = .065, *d* = 0.35). Response biases did not differ significantly between the groups (SSDT*: p* = .782; cvSDT: *p* = .743). However, higher somatic symptom distress was significantly associated with a more liberal interoceptive response tendency in both tasks in the sham EMF group (two significant models, one trend: (-0.209 ≤ *β*s ≤ -0.325, adjusted 0.232 ≤ *R*² ≤ 0.330).

**Conclusions:**

A liberal approach was associated with elevated symptom experience across bodily domains and might be considered a transdiagnostic psychopathological risk factor. As research is still scarce, replication studies with valid context manipulations are essential.

## Introduction

Changes in interoceptive processes are considered a core factor contributing to the development and maintenance of mental disorders associated with elevated somatic symptom distress (Henningsen et al., 2018; Krautwurst et al., 2016; Witthöft et al., 2020; Wolters et al., 2022). Symptom perception can be understood as an active inference account of interoceptive sensations, in which predictions on symptoms are compared to the somatosensory input itself at different levels of perception (Köteles, 2021; Van Den Bergh et al., 2017). According to the predictive processing theory, the brain constantly makes assumptions about the causes of incoming sensory input based on prior experiences and knowledge (so-called priors). These priors are neuronally represented as probability distributions. A discrepancy between a prior and the current sensory input leads to a prediction error. One way to minimize prediction errors and optimize perception is the refinement of existing hypotheses (e.g., updating priors by changing expectations; Barrett & Simmons, 2015). However, when somatosensory input is vague and (symptom) priors are strong, perceptions may align more with the priors than with the somatosensory input, leading to a persistent disconnect between perception and physiology. Priors will not be updated accordingly and symptoms persistently perceived (Ainley et al., 2016; Barrett et al., 2016; Van Den Bergh et al., 2017).

To better understand this decoupling in somatic symptom distress we can empirically assess decision strategies during perceptions of internal bodily signals (Petersen et al., 2015; Pohl et al., 2021; Van Den Bergh et al., 2021b; Wolters et al., 2022). One can think of these strategies as an inner “alarm system” that ranges from conservative (“wait and see”) to liberal (“better safe than sorry”; Lynn & Barrett, 2014). A liberal approach is associated with a more sensitive “alarm system”, where weak bodily signals are more likely to be detected and the number of false alarms (illusory perceptions) increases. This reduces the probability of missing a bodily signal but bears the risk of mistaking ambiguous, however harmless body sensations as symptoms of a disease (Petersen et al., 2015; Wolters et al., 2022). Examining the influence of contextual information on the decision strategy is a promising way to test predictive processing framework assumptions: Threat-related contexts, in which body sensations are predicted should lead to a liberalization in the response strategy and decouple perception from physiological input. Such contexts can be created in the laboratory using sham electromagnetic field (EMF) exposure. Laboratory studies show that individuals who describe themselves as hypersensitive to EMF report various somatic symptoms and mental distress under sham EMF exposure (e.g., Eltiti et al., 2018; Schmiedchen et al., 2019). Also, analogue samples rate tactile stimuli as more intense and report diverse bodily symptoms such as dizziness, tingling, headache, muscle shivering or loss of concentration when expecting EMF exposure (Bräscher et al., 2017, 2020; Eltiti et al., 2018; Szemerszky et al., 2010; Verrender et al., 2018; Witthöft & Rubin, 2013; Wolters et al., 2021).

Furthermore, sham EMF leads to an increase in "illusory touch experiences" and a liberal response bias as measured with the so-called Somatic Signal Detection Task (SSDT; Lloyd et al., 2008; Wolters et al., 2021). The SSDT assesses the tendency to experience vibrotactile sensations in the absence of somatic input in a laboratory setting (Brown et al., 2010, 2012; Katzer et al., 2011; Lloyd et al., 2008; Wolters et al., 2021). Meta-analytic evidence supports a liberalization of tactile perceptions in somatic symptom and related disorders using this task (Wolters et al., 2022). Importantly, liberalization of responses was also shown under sham Wi-Fi exposure (Wolters et al., 2021). Apart from vibrotactile perception, it remains unclear whether sham EMF liberalizes bodily signals from within the body. In the past decades, interoception research has focused primarily on the cardiovascular body domain, as the heartbeat is a continuous bodily signal that can be easily measured in a laboratory setting (Garfinkel et al., 2022; Phillips et al., 1999). A key finding of this research is that most people struggle to accurately perceive their heartbeat at rest (Brener & Ring, 2016; Köteles, 2021; Köteles et al., 2020; Zamariola et al., 2018). It is assumed that the heartbeat represents a weak bodily signal for most people in everyday life (except for physical activity or emotional states; Köteles, 2021). Therefore, priors, rather than an accurate perception of one’s heartbeat, seem crucial for cardiac interoception (Körmendi et al., 2021; Parrotta et al., 2024). The cardiovascular Signal Detection Task (cvSDT) constitutes a novel measurement which assesses sensitivity towards the own heartbeats *and* response strategies (Pohl et al., 2021). In the cvSDT participants are asked to silently count their heartbeats and to decide whether a presented interval (e.g., 6–8) or an alternative answer (less/more) is correct.

The current study aimed to (1) replicate previous findings showing that symptom-provoking context information (sham EMF) led to a liberalization of response tendencies in tactile perceptions (Wolters et al., 2021). Importantly, we also sought to (2) assess whether this manipulation affects response tendencies across different body domains. We believe that several aspects of our study advance the understanding of the mechanisms underlying somatic symptom burden. A key open question is whether interoceptive response biases are domain-specific or generalizable across bodily modalities. Whereas vibrotactile stimuli are applied externally to the skin surface, the heartbeat constitutes an internal bodily sensation. To our knowledge, context dependent changes in response strategies have not yet been investigated for signals originating from within the body itself. We propose that response biases related to internal bodily sensations are particularly relevant to the development and maintenance of pathological somatic symptom perception. The heartbeat, in particular, is a bodily signal closely linked to potentially threat-related bodily symptoms (e.g., heart racing, palpitations). In the presence of objective disease indicators, missed detections may entail relevant health-related costs (e.g., coronary heart disease), whereas a “better-safe-than-sorry” approach can reduce such costs (Van den Bergh et al., 2017). However, in the absence of physiological pathology, a liberal perceptual bias may contribute to increased symptom perception and the emergence of persistent somatic symptoms (PSS; Van den Bergh et al., 2017). We compared two groups of participants in a between-subject design: In the sham EMF group, participants were told that they were exposed to a strong EMF while completing the SSDT and the cvSDT (sham EMF). In the control group, participants were told that EMF radiation was turned off (sham EMF off). We assumed that participants in the sham EMF group would report more bodily sensations and show a more liberal response bias in the tactile and cardiac body domain than participants in the control group. A positive correlation between the response bias scores in the cvSDT and the SSDT was expected. In line with previous findings suggesting a direct influence of the (prior) expectation on response bias but not on sensitivity (Bang & Rahnev, 2017), we did not expect participants in the sham EMF group to show higher sensitivity in these two domains of bodily perception.

## Method

The study protocol was preregistered at OSF (https://doi.org/10.17605/OSF.IO/S6WU2) and approved by the ethics committee of the Faculty of Human Sciences at the University of Cologne on 28 August 2021 (APHF0126). The study is in accordance with the *Declaration of Helsinki,* and all procedures were performed in compliance with relevant laws and institutional guidelines. Data and analysis code are available at https://osf.io/rswve).

### Sample

One hundred and thirteen participants were recruited exclusively to take part in the present experiment (76.1 % female, 0 % diverse). The mean age was 25.12 years (range of 18–52, *SD* = 5.79). The study was conducted in the laboratory of the Department of Clinical Psychology and Psychotherapy at the University of Cologne. Participants were recruited via online announcements on different websites of the university, mailing lists, and posters at the university campus. As part of the cover story, the advertisement said that the study investigated the effects of electromagnetic radiation on different types of bodily perception. Exclusion criteria included current or previous psychotic or substance use disorders, chronic diseases such as diabetes, prior neurological diseases such as a concussion, intake of medication that might influence perception, other severe physical disabilities, pregnancy, or current breastfeeding. All participants received either reimbursement (8.50€ per hour) or course credit.

An a-priori power analysis was conducted with G*Power for a MAN(C)OVA with two groups and one covariate (df = 1; Faul et al., 2007). The power analysis was based on the results of a previous study by Wolters and colleagues (Wolters et al., 2021), who found a medium effect size for the influence of sham EMF on liberalization of responses in the SSDT. A required sample size of 93 participants was indicated for an effect size of *d* = 0.30 at an alpha level of 0.05 and 80 % power. Data collection was carried out between 06/12/2021 and 29/04/2022.

### Procedure and cover story

Before the experiment, a short telephone interview was conducted to check whether participants met the study criteria. Eligible participants were invited to the laboratory and asked not to drink alcohol for 8 h and coffee for 2 h before the appointment.

In accordance with legal requirements during the Corona pandemic, the investigators and participants wore FFP2 masks. The cover story followed the study protocol of Wolters and colleagues (Wolters et al., 2021): A Wi-Fi symbol with a warning sign was placed on the door of the lab. The computer screen was surrounded by two Wi-Fi signal repeaters pointing in the direction of the participants face. After participants provided written informed consent, they were either told that they would be exposed to enhanced EMF radiation (sham EMF group) or that EMF repeaters were switched off (control group). The group assignment was pseudo-randomized. The SSDT and the cvSDT were completed consecutively in pseudo-randomized order. The SSDT was completed with the index finger of the dominant hand. Before entering the lab, participants in the sham EMF group were asked to either switch their phones off or into flight-mode. Two dummy Wi-Fi repeaters were switched on so that they were blinking. Participants were informed that the apparatus was built in cooperation with the Department of Physics and that the radiation was twice as high as a normal EMF radiation. A safety plug connector was switched on and a Wi-Fi symbol appeared on the iPod screen. The experimenter informed the participants that she would leave the room during the experiment due to strong EMF radiation. Participants were told that perception of tactile stimulation might be increased during EMF radiation and that the EMF radiation could cause mild symptoms such as headache, dizziness, or tingling, but no severe or long-term complaints. After both tasks were completed, all participants answered questionnaires on the computer and were debriefed.

### Demographics and questionnaires

Participants were asked to provide the following demographic information: age, height and weight. The Edinburgh Handedness Questionnaire was used to determine participants' dominant hand. The amount of physical activity per week was measured with the Brief Physical Activity Assessment (Marshall et al., 2005). Anxiety levels/negative affectivity were assessed via the State-Trait Anxiety Inventory (Form X) on a Likert scale from 1 (not at all/almost never) to 4 (very much so/almost always; STAI-S, Cronbach’s α = 0.896 and STAI-T, Cronbach’s α = 0.918 in the present study; Spielberger, 2012). Each STAI subscale consists of 20 self-report items and a sum score with a range from 20–80 is calculated separately for each subscale. Somatic symptom distress was assessed using the 15-item Patient Health Questionnaire (PHQ-15, Cronbach’s α = 0.616; Kroenke et al., 2002). Levels of somatic symptom severity are measured on a 3-point Likert scale from 0 (“not bothered at all”) to 2 (“bothered a lot”). The short version of the Sensitive Soma Assessment Scale (SenSo-EMF, Cronbach's α = 0.941 in the present study) measured sensitivity to EMF on a 5-point Likert scale from 1 (“strongly disagree”) to 5 (“strongly agree”; Nieto-Hernandez et al., 2008). The Senso-EMF total score ranges from 5–25. A self-developed, short checklist on symptom experience (CSE) was used to retrospectively assess different bodily symptoms during the experiment (e.g., tingling, tension, vibration, or shaking of the body) on a 5-point Likert scale on a range from 1 (“not at all”) to 5 (“very strong”). The sum score ranges from 5–45. A debriefing questionnaire served as a manipulation check. Participants were asked to indicate their level of conviction (in %) about the exposure to elevated EMF radiation (sham EMF group) or non-elevated EMF radiation, respectively (control group). Participants in the sham EMF group were also asked to indicate their worries concerning the EMF exposure (in %: "How strong was your worry/concern about the Wi-Fi exposure during the experiment?") and possible effects of EMF radiation on their bodily perception (open response format).

### Tactile stimulation

Vibrotactile stimuli were delivered to the index finger of the participant’s dominant hand using a 1.4 × 2.3 bone conductor (Oticon BC461–1). Tactile stimulation consisted of a 50 Hz sinusoidal wave. The bone conductor and a light diode were built into a 13 × 17 cm plastic pad. Directly below the light diode, an iPod was fixed to present experimental instructions and keep participants’ attention on their hand. Participants wore on-ear headphones to prevent them from hearing the vibrations and to present acoustic signals during the experiment.

To measure individual tactile thresholds, participants had to indicate whether they felt a vibration in the first or second of two consecutive intervals. A 3-down-1-up procedure (Levitt, 1971) with a two-alternative forced-choice (“yes”/”no”) response format was used. One interval lasted 2300 ms and a vibrotactile signal was randomly presented for 20 ms in the first or second interval after 600 ms. To get used to the SSDT, five test trials were conducted. The thresholding procedure started with an intensity that most people perceive without problems (50 arbitrary units; range: 0–255). Each incorrect choice led to an increase of vibration intensity and three correct answers to a decrease. After 6 reversals with an increase/decrease of 3 arbitrary units, the change in intensity was more fine-graned for the last 4 reversals (1 arbitrary unit). The tactile threshold was indicated by the signal intensity presented at last. Vibration strength was adjusted until participants perceived approximately 79.4 % of trials (Levitt, 1971). Tactile thresholds can be measured reliably using this procedure (*r* = 0.761; Wolters et al., 2021).

The main task consisted of 80 trials. In each trial, participants had to indicate whether they felt a vibration (“yes”/”no”). Four conditions were each displayed 20 times in randomized order: vibration-only, vibration-and-light, light-only, and no-stimulus. The start and end of each trial was marked by a beeping tone. The duration of trials was 2.3 s. Vibrations were presented in the middle of the trial for 20 ms. The vibration intensity was based on the determined individual thresholds. In the vibration-and-light condition, LED was presented simultaneously with the vibration.

### Electrocardiogramm measurements

The Varioport system (Becker, Meditec, Karlsruhe, Germany) was used to record the electrocardiogram ECG. ECG electrodes were attached below the left costal arch and on the left and right clavicle. The detection of R-waves was based on an algorithm by Vary (1980). To run the experiment and analyze biodata, the custom-built software UVariotest (programmed by Gerhard Mutz, University of Cologne) was used. Before the cvSDT, participants were asked to relax and sit still for one minute, while the baseline heart rate was assessed. Participants were then instructed to silently count their heartbeats during trials. The number of consecutive heartbeats (i.e., R-spikes) determined the length of each trial. One trial lasted five to 13 heartbeats, five target intervals with differing length were presented (6–8, 7–9, 8–10, 9–11, 10–12). Before each trial, the instruction “Lean back relaxed and count all heartbeats that you can sense after you hear the starting tone” appeared for 1.5 s. The trial then started with the next R-wave triggering a tone as starting signal. The requested number of R-waves was counted by the online algorithm and the stop signal was initiated by the last R-wave and audible with an average delay of 90 ms. After 500 ms, participants were asked to indicate whether a presented interval was correct or more/less hearbeats were counted in a two-alternative forced-choice response format. Following the procedure of Pohl and colleagues (2021), participants could either choose between a presented target interval (e.g., 7–9) or the alternative presented as “less”/”more”. In 50 % of the cases, the interval option was correct and in the other 50 % of the cases, the alternative option (“less”/”more”) was correct. The “less” category was shown 40 times (i.e., 4 times each length in each of the 2 conditions: “interval correct”, “alternative correct”) and the “more” category was shown 10 times as a distractor. Interval lengths and conditions were presented in random order for each participant. In sum, the task consisted of 4 test trials (one for each condition) and 50 experimental trials and lasted approximately 10 min.

### Data analysis

Data preparation and analysis were performed with R (R Core Team, 2024) and SPSS 28.0 (IBM SPSS Statistics for Windows, 2021). The outcomes of the SSDT and cvSDT were analyzed separately based on signal detection theory (Macmillan & Creelman, 1990). ECG data were visually inspected for artifacts due to movements, extrasystoles, or other noise. Eight participants had to be excluded from the data analysis of the cvSDT due to serious ECG abnormalities.

#### Analyses of SDT parameters (cvSDT/SSDT)

Correctly identified signals were categorized as hits (i.e., in the SSDT a “yes” response whenever a vibration was present, in the cvSDT choosing the interval whenever the interval was correct). Falsely identifying a signal as present when it was actually absent was categorized as a false alarm (i.e., in the SSDT a “yes” response in trials without vibrations, in the cvSDT choosing the interval although less heartbeats were recorded). Hit and false alarm rates were calculated and log-linear corrected following the method outlined by Snodgrass and Corwin (1988):

hits=[(hits+0.5)/(hits+misses+1)];
false alarms=[(false alarms+0.5)/(false alarms+correct rejections+1)].
The following formula was used to calculate the sensitivity index:
*d’*=(z[hits]–z[false alarms]).
Response bias c was calculated with the formula:
*c*=(−0,5×[z(hits)]+z(false alarms)).

In the cvSDT, z-scores of corrected hits and false alarms were computed with a loglinear transformation to calculate the indices *d’* and *c* (Appendix in Macmillan & Creelman, 2005). The value of *c* ranges between −2.33 and +2.33. The range of *d’* usually lies between zero and approximately 4.65, whereby a higher score indicates higher sensitivity. A negative value of *c* represents a liberal bias, while a positive value indicates a conservative response bias (Macmillan & Creelman, 1990).

#### Hypothesis based analyses

A multivariate variance analysis (MANOVA) was conducted to assess differences between groups for demographics, debriefing, and symptom-related questionnaire outcomes. Since the homogeneity of error variances was not met (Levene’s test: *p* < .05), the MANOVA was repeated after applying the Box-Cox transformation (Box & Cox, 1964) to three variables: BMI, symptom reporting, and the debriefing questionnaire.

For the SSDT, effects on *c* and *d’* were analyzed using two separate AN(C)OVAs with repeated measures (LED/no-LED). In each of the AN(C)OVAs, the order of the two tasks (SSDT and cvSDT) was first included as a covariate. Effects on sensitivity and response bias in the cvSDT were analyzed in a single MAN(C)OVA with order included as covariate. Task order was excluded from the respective statistical model if it did not show significance.

Correlations of response bias *c* in the cvSDT and the SSDT were calculated for each group separately (EMF/no-EMF). For all analyses, data were checked for outliers.

#### Exploratory analyses

We conducted four multiple regression analyses for both groups to examine whether response bias *c* in the SSDT and the cvSDT explained variance in i) somatic symptom distress (PHQ-15) and ii) symptom experience during the experiment (CSE). Trait anxiety was included in the PHQ models and state anxiety in the CSE models as covariate (compare Pohl et al., 2021). Both predictors were added simultaneously using the “enter” method.

Homoscedasticity (tolerance value < 0.1) and multicollinearity (VIF > 1) were checked for all models. Outliers and influential cases were identified using casewise diagnostics, average leverage, Mahalanobis Distance, and Cook’s distance. Cases deviating in at least two outlier statistics were excluded from the respective regression model.

## Results

### Manipulation check and group characteristics

There were significant differences between the groups, *V* = 0.547, *F*(9, 103) = 13.800, *p* < .001, *d* = 2.20. Conviction levels about an elevated EMF radiation during the experiment were significantly higher in the sham EMF group than in the control group (60.86 vs. 9.88), *F*(1111) = 107.860, *p* < .001, *d* = 1.97. Participants in the control group (*n* = 56) reported a median conviction of 1 % (IQR = 50) that they had been exposed to enhanced EMF radiation. Two controls indicated that they were >50 % convinced that EMF radiation was turned on. Median conviction was 70 % (IQR = 50) in the sham EMF group (*n* = 57). Approximately one-quarter (22.8 %) of participants was skeptical about the EMF exposure (rating of 25 % or lower). More than half of the participants in the sham EMF group (57.9 %) were >50 % convinced of the EMF exposure and about one-quarter (26.3 %) of participants in the sham EMF group reported high levels of conviction (> 90 %). Higher conviction levels in the sham EMF group were associated with higher worry about the effects of EMF radiation (*r* = 0.270, *p* = .042).

Median worry about the effects of EMF radiation was 5 % (IQR = 50) in the sham EMF group. More than one-third of participants (38.6 %) reported no worry (1 %) about EMF radiation (note: 1 % was the lowest value to choose). A manipulation check on the association between worry about EMF and symptom perception was performed. To this end, participants in the sham EMF group were divided into two groups (no worries reported vs. worries reported). Participants in the sham EMF group who reported worries about EMF (> 1 %, *n* = 35) experienced significantly more symptoms during the experiment than participants in the control group (*n* = 56), *p* = .018, *Mdiff* = 2.60, 95 %-CI[−4.75, −0.45]. No significant difference in symptom perception was found between participants in the sham EMF group, who reported no worry about EMF (*n* = 22), and controls, *p* = .587, *Mdiff* = −0.69, 95 %-CI[−3.20, 1.82].

Participants in the sham EMF group reported trend-wise more symptoms during the experiment (*F*(1111) = 3.477, *p* = .065, *d* = 0.35) and significantly higher levels of state anxiety (*F*(1111) = 5.520, *p* = .021, *d* = 0.44) than controls. No other significant group differences were found, *p* > .05 (see Table 1).

**Table 1:** Descriptive Statistics and Group Differences for Questionnaire Data.

		Control group (*n* = 56)			Sham EMF group (*n* = 57)				
	*M*	*Range*	*SD*	*M*	*Range*	*SD*	*F*	*p*	*d*
Age	24.84	18–42	5.54	25.46	18–52	6.04	.320	.573	0.11
BMI	23.38	16.87–40.30	4.34	22.14	17.01–29.35	2.49	1.962	.164	0.09
Physical Activity	4.30	0–8	2.29	4.12	0–8	2.32	.174	.678	0.26
PHQ-15	6.82	0–15	3.52	7.32	1–15	3.11	.628	.430	0.16
CSE	13.95	9–32	4.23	15.81	9–37	5.75	3.477	.065	0.35
SenSo-EMF	8.09	5–15	3.55	9.25	5–20	3.93	.104	.104	0.31
STAI-S	35.63	20–57	7.78	39.33	24–63	8.95	5.520	.021	0.44
STAI-T	39.32	22–61	10.08	39.68	25–68	10.15	.036	.849	0.04

CSE = Checklist on Symptom Experience, PHQ-15 = Patient Health Questionnaire, SenSo = Sensitive Soma Assessment Scale, EMF version, STAI-*S* = State-Trait Anxiety Inventory, state version, STAI-*T* = State-Trait Anxiety Inventory, trait version.

### Response bias and sensitivity in both groups

For both experimental tasks, descriptive statistics of SDT indices (*c* and *d’*), hit rates and false alarm rates are displayed in Table 2.

**Table 2:** Descriptive Statistics for Hit Rates, False Alarm Rates, Response bias c and Sensitivity d’ for both tasks and groups.

	Control group Mdn (IQR)		Sham EMF group Mdn (IQR)	
	SSDT	cvSDT	SSDT	cvSDT
Hit rate ( %)	58.33 (44.05)	35.71 (38.10)	61.90 (40.48)	33.33 (36.90)
FA rate ( %)	7.14 (6.55)	7.14 (9.52)	7.14 (9.52)	4.96 (9.52)
Response bias *c*	0.57 (0.54)	0.04 (0.54)	0.63 (0.78)	0.12 (0.54)
Sensitivity *d’*	1.71 (1.62)	1.03 (0.89)	1.74 (1.21)	0.88 (0.79)

cvSDT = cardiovascular Signal Detection Task, FA = False Alarm, IQR = Interquartile Range, Mdn = Median, SSDT = Somatosensory Signal Detection Task.

#### SSDT

Thresholds did not differ significantly between the groups, control group: *M* = 33.55 (*SD* = 23.66), sham EMF group: *M* = 33.84 (*SD* = 23.82), *t*(111) = −0.065, *p* = .949, *d* = −0.01. There was a significant effect of LED on both indices, *F*(1, 111) = 32.986, *p* < .001, *d* = 1.09 for *c* and *F*(1, 111) = 17.335, *p* < .001, *d* = 0.79 and *d’*. In trials with a simultaneous light stimulus, sensitivity index *d’* was higher *M* = 1.73 (*SD* = 1.04) vs. *M* = 1.47 (*SD* = 0.96) and the response bias *c* was more liberal *M* = 0.50 (*SD* = 0.60) vs. *M* = 0.76 (*SD* = 0.53) compared to light-off trials. Hit rates and FA rates as well as *d’* and *c* of all SSDT trial types are depicted in S1 Table. No significant group difference was found for *c* (*F*(1, 111) = 0.077, *p* = .782, *d* = 0.06) and *d’* (*F*(1, 111) = 0.041, *p* = .840, *d* = 0.03). There was no significant effect of the order of tasks on response bias *c* (*F*(1, 110) = 0.052, *p* = .820, *d* = 0.04) and sensitivity index *d’* (*F*(1, 110) = 0.438, *p* = .510, *d* = 0.13).

#### cvSDT

There was no significant difference between the groups for *c* (*F*(1, 103) = 0.108, *p* = .743, *d* = 0.06) and *d’* (*F*(1, 103) = 0.610, *p* = .437, *d* = 0.15). The order of tasks showed no significant effect on the response bias *c* (*F*(1, 101) = 0.548, *p* = .461, *d* = 0.14) and the sensitivity index *d’* (*F*(1,101) = 0.082, *p* = .775, *d* = 0.06).

#### Response bias among bodily domains

No significant correlation was found for response bias *c* in the SSDT or the cvSDT, in either the sham EMF group (*r* = 0.052, *p* = .713) or in the control group (*r* = 0.106, *p* = .453).

### Exploratory analyses

#### General somatic symptom distress

In both the sham EMF group and the control group, trait anxiety (STAI-T) significantly predicted general somatic symptom distress (PHQ-15), whereas *SSDT* response bias did not. Specifically, in the sham EMF group, trait anxiety (*β* = 0.363, *p* = .006) was a significant predictor of general somatic symptom distress (PHQ-15), *F*(2, 53) = 4.089, *p* = .022, adjusted *R²* = 0.101, while *SSDT* response bias was not (*β* = 0.037, *p* = .775, see S2 Table). Similarly, in the control group, trait anxiety (*β* = 0.372, *p* = .006) significantly predicted somatic symptom distress (PHQ-15), *F*(2, 52) = 4.543, *p* = .015, adjusted *R²* = 0.116, with *SSDT* response bias showing no significant predictive value (*β* = 0.059, *p* = .650, see S3 Table).

In the sham EMF group, both *cvSDT* response bias (*ß* = −0.325, *p* = .014) and trait anxiety (*ß* = 0.420, *p* = .002) significantly predicted somatic symptom distress (PHQ-15): *F*(2, 46) = 8.243, *p* < .001, adjusted *R²* = 0.232 (see S4 Table). However, in the control group, only trait anxiety (STAI-T, *ß* = 0.414, *p* = .002), but not the *cvSDT* response bias (*ß* = 0.060, *p* = .647) predicted somatic symptom distress (PHQ-15): *F*(2, 49) = 5.264, *p* = .008, adjusted *R²* = 0.143 (see S5 Table).

#### Experimentally induced somatic symptoms

In the sham EMF group, somatic symptoms during the experiment (CSE) were significantly predicted by both the *SSDT* response bias (*β* = −0.262, *p* = .024) and state anxiety (STAI-S: *β* = 0.488, *p* < .001), *F*(2,53) = 14.546, *p* < .001, adjusted *R²* = 0.330 (see S6 Table). In contrast, the control group showed no significant prediction of somatic symptoms during the experiment (CSE) by either state anxiety (*β* = 0.150, *p* = .294) or *SSDT* response bias (*β* = −0.019, *p* = .893), *F*(2, 50) = 0.563, *p* = .573, adjusted *R²* = −0.017 (see S7 Table).

Moreover, in the sham EMF group, there was a trend for the *cvSDT* response bias predicting experimentally induced symptoms (*β* = −0.209, *p* = .094), while state anxiety significantly predicted these symptoms (*β* = 0.502, *p* < .001), *F*(2, 46) = 11.429, *p* < .001, adjusted *R²* = 0.303 (see S8 Table). However, in the control group, neither *cvSDT* response bias (*β* = 0.040, *p* = .779) nor state anxiety (STAI-S, *β* = 0.146, *p* = .311) predicted somatic symptoms during the experiment, *F*(2, 49) = 0.621, *p* = .542, adjusted *R²* = −0.015 (see S9 Table), underscoring a clear distinction between the two groups. In summary, the variance explained by anxiety and response bias ranged from 13.4 % to 33 % in the sham EMF group and from 0 % to 14.3 % in the control group. Notably, in three out of four models, a more liberal response style was associated with an increased somatic symptom experience in the sham EMF group, whereas no such association was found in the control group.

## Discussion

The primary aim of this study was to investigate the influence of contextual, threat-related information on interoceptive processes across different body domains. We expected that participants in the sham EMF group would show a more liberal response bias than those in the control group for both tactile and cardiovascular perceptions, and that the response bias would correlate across the two tasks. Regarding the SSDT, tactile thresholds were comparable to a previous study (Wolters et al., 2021). In line with previous research, participants showed higher sensitivity scores and applied a more liberal response bias in light-present trials in this task (Katzer et al., 2011, 2012; Wolters et al., 2021). As expected, no group difference was found for sensitivity in both tasks. Contrary to our assumptions, the sham EMF condition was not associated with a liberalization of response tendencies in either of the SDT tasks in our study. However, in addition to anxiety, somatic symptom distress was significantly predicted by more liberal responses in two and trendwise in one of four regression models under sham EMF.

These findings are of particular interest, as negative affect is regarded as a main factor for symptom experience in sham EMF studies and for somatic symptom burden in general (Szemerszky et al., 2010; Van Den Bergh et al., 2017; Van Den Bergh & Walentynowicz, 2016; Van Den Houte et al., 2017; Witthöft & Rubin, 2013). Importantly, the connection between somatic symptom experience and liberal response bias was observed across bodily domains.

The lack of group difference in response bias is likely attributable to the reduced effectiveness of the cover study. First, the conviction of the cover story in our study was lower in comparison to a previous study with a similar study design (65 % vs. 90 %; compare Wolters et al., 2021). Second, participants in the sham EMF group reported elevated levels of state anxiety, but concern about the consequences of electromagnetic radiation was low (5 % vs. 12.25 %; in comparison to Wolters et al., 2021). However, self-reports showed a trend towards increased symptom perception during the experiment in the sham EMF group which reached significance in participants worried about the EMF radiation. Thus, the experimental design seems appropriate to investigate changes in bodily perception associated with adverse health expectations (compare Bräscher et al., 2017, 2020; Wolters et al., 2021).

Interestingly, under sham EMF a liberal response bias in the cardiovascular domain corresponded with elevated somatic symptom distress in our study, while no significant relation between these was found in the cvSDT without any experimental manipulation (Petzke et al., 2024; Pohl et al., 2021). Moreover, the observed association between symptom reports during the experiment and a liberal response bias in the SSDT supports this interpretation. This suggests that we were able to demonstrate a liberalization of response strategy among those participants for whom the cover story was effective (i.e., who experienced symptoms). The results are in line with previous studies showing associations with elevated levels of somatic symptom distress (PHQ-15) and health anxiety in this task (Katzer et al., 2011, 2012). However, our study revealed no association between the PHQ-15 and liberal response bias in the SSDT for the sham EMF group. A recent study incorporating a sham EMF condition as a within-subject factor provided empirical evidence in favor of the predictive processing theory, demonstrating the influence of prior expectations about adverse health effects on the somatosensory response bias (Wolters et al., 2021). Additionally, cardiac interoceptive illusions (i.e., overreporting of heartbeats) were elicited in a non-clinical sample when participants expected to receive a painful stimulus, which indicates that threatening contexts and a certain expectation (i.e., increase of heart rate) can cause biases in cardioception (Parrotta et al., 2024). Heart-related sensations (e.g., chest pain, palpitations) often form a starting point for general medical assessment, although a physical cause is rarely found (Katon, 1996; Richards, 1992). Individuals with high somatic symptom burden (e.g., patients suffering from somatic symptom disorder or anxiety-related disorders) regularly report distressing heart-related sensations and heart-focused anxiety due to the potentially serious negative consequences (e.g., heart attack or sudden cardiac death; Eifert et al., 2000). Therefore, we consider the cardiovascular body domain to be of particular relevance for the investigation of somatic symptom burden. It is assumed that decision strategies depend on contexts shaping prior expectations (Lynn & Barrett, 2014; Wolters et al., 2021, 2022). In case individuals expect high threat (e.g., adverse health effects) and experience uncertainty (e.g., highly ambiguous body sensations), body perception presumably gets liberally biased (Wolters et al., 2022). This “better safe than sorry” approach is adaptive when it is linked to objective evidence of disease factors and missing or misclassifying a bodily sensation as harmless would cause negative outcomes for the individual such as adverse health effects (Van Den Bergh et al., 2017; Van Den Bergh et al., 2021b). In case of rigid and overly precise threat-related predictions, perception presumably becomes decoupled from physiological function and liberalization forfeits its adaptive effect (Henningsen et al., 2018; Lynn & Barrett, 2014; Van Den Bergh et al., 2017; Van Den Bergh et al., 2021b; Wolters et al., 2022). Thus, PSS could be the result of an initially functional processing strategy that, over time, lacks differentiation and becomes biased towards illness and threat (Henningsen et al., 2018; Van Den Bergh et al., 2017). A positive relationship between somatic symptom distress and liberality in response behavior across different bodily domains (i.e., tactile perceptions and perception of spontaneous skin conductance fluctuations) has been identified in a meta-analytic review (Wolters et al., 2022). Therefore, liberally biased perception can be assumed as a risk factor for somatic symptom burden and a promising factor in the investigation of underlying psychopathological mechanisms of PSS, respectively (Van Den Bergh et al., 2021b; Wolters et al., 2021, 2022).

Our findings support these assumptions and suggest that it could be important to specifically address the aspect of threat-related top-down priors and response biases in the treatment of PSS. Symptom provocation could support the adaptation of interoceptive representations of the body, on the premise that patients adopt an acceptance attitude and abandon dysfunctional control strategies (e.g. body checking or body scanning; Henningsen et al., 2018).

### Study limitations and future directions

There is an ongoing debate about suitable measures of the capability to accurately sense interoceptive signals, particularly concerning heartbeat perception (Ainley et al., 2020; Corneille et al., 2020; Murphy, 2024; Tsakiris et al., 2019; Zimprich et al., 2020). Signal detection tasks can overcome some of the limitations by disentangling sensitivity (“interoceptive accuracy”) and response bias. Although both the cvSDT and the SSDT measure ambiguous bodily signals, hit rates were lower in the cardiovascular domain than in the somatosensory domain in our study (58.33 % in the SSDT vs. 35.71 % in the cvSDT in the control group, and 61.90 % vs. 33.33 % in the sham EMF group). This is consistent with previous findings on cardioceptive accuracy, which demonstrate that many individuals have difficulties in accurately perceiving their heartbeats (Brener & Ring, 2016; Körmendi et al., 2022; Zamariola et al., 2018). Nevertheless, the discrepancy in hit rates presumably accounts for the lack of correspondence between the response biases of the two tasks. We propose two potential solutions to address this discrepancy: 1) the strength of the cardioceptive signals measured via the cvSDT could be increased, for example through heightened arousal (e.g., stress tasks), or 2) the threshold for vibrotactile stimuli in the SSDT could be lowered to render these sensations more ambiguous. We regard the investigation of two bodily domains using the same experimental framework (i.e., signal detection tasks) as a valuable strength of our study and believe that this approach holds considerable potential for future research on psychopathological mechanisms of PSS (Lynn & Barrett, 2014). The assessment of interoceptive processes across body domains using tasks with comparable cognitive and perceptual demands remains rare (Harver et al., 1993; Whitehead & Drescher, 1980). As the cvSDT is a novel method, research on interoceptive processes measured by this task is still scarce. In addition, the presentation of the start tones, which were coupled to the participants' R-waves, proved to be suboptimal and should be improved in future studies using this measure. The results presented here should therefore be interpreted with caution. Hence, further studies are needed to investigate convergent validity across SDT tasks.

The results of our exploratory analyses are consistent with theoretical assumptions and previous findings (e.g., Wolters et al., 2021, 2022). However, we conducted four exploratory regression models, each with two predictors for each group. It is well known that multiple tests increase the risk of false positive results. Therefore, our findings should be seen as preliminary and hypothesis-generating. A preregistration of hypotheses regarding the relationship between self-reported somatic symptom burden and response bias is highly recommended for future studies. Another aspect that merits attention in future studies is the measurement of negative affectivity. A recent meta-analysis suggests that the STAI-T presumably captures the broad trait of negative affectivity (Knowles & Olatunji, 2020). Notably, the STAI-T is still the most widely used measure of trait anxiety. However, for future studies, the Positive and Negative Affect Schedule (PANAS) could be a useful additional measure in the investigation of associations with somatic symptom distress as it covers a wider range of (negative) emotional states (e.g., guilt, shame and hostility; Knowles & Olatunji, 2020).

The PHQ-15 showed relatively low internal consistency in our study, which might have impeded the results (compare Katzer et al., 2011). Due to the low internal consistency and the fact that hypotheses conform findings stem from exploratory analyses, further experimental and longitudinal studies are needed for replication and to examine the cause-effect relationship and the long-term effects of adverse health expectations and response bias.

Although no significant group difference in symptom experience was found between the sham EMF and control group during the experiment, participants in the sham EMF group who were more worried about EMF radiation reported a higher number of symptoms. Therefore, finding ecologically valid manipulations of threatening contexts for experimental studies will be of utmost importance to examine biased interoception. Although verbal suggestions can be sufficiently convincing, the use of addtional (pseudo-)scientific material like television reports, newspaper articles or images could further increase concern and probably lead to adaptions in response bias (compare Bräscher et al., 2017; Wolters et al., 2021).

In general, increased symptom reporting due to contextual information depends on personal characteristics (Van Den Bergh et al., 2017). Anxious people and individuals who tend to attribute symptoms to external causes exhibit higher symptom reporting in sham EMF conditions (Van Den Bergh et al., 2021a; Witthöft & Rubin, 2013). The influence of individual characteristics suggests that a homogeneous sample may not be ideal for answering the research question at hand. As we have investigated interoceptive processes in an analogue sample consisting mainly of psychology students, further investigation in clinical samples (e.g. patients with increased somatic symptom burden, health anxiety or idiopathic environmental intolerances) is crucial to elucidate underlying psychopathological mechanisms. A preselection procedure, such as a short screening using self-report measures (e.g., PANAS, PHQ-15, SenSo-EMF) could be employed in future studies to identify individuals who are more prone to experience health-related threats (compare Brown et al., 2010).

### Conclusions

According to the predictive processing theory, overly precise priors, represented as threat-related expectations, can lead to a decoupling of interoceptive perception and sensory input. This effect is amplified when the sensory input is imprecise or ambiguous. Liberal response behavior, the overestimation of bodily symptoms, reflects this decoupling process. Our study did not replicate a previous study which demonstrated a liberal response bias for the somatosensory domain induced by sham EMF. Additionally, there was no group difference in response bias in the cardiovascular domain. However, self-reports of symptom experience were trendwise elevated, and somatic symptom experience was associated with liberal response bias across bodily domains in the sham EMF group. This is consistent with recent findings and assumptions that a “better safe than sorry” strategy may underlie general psychopathology, including somatic symptom distress. In this sense, our study can be seen as a valuable contribution to a broader understanding of the (predictive processing) mechanisms involved in interoception and, in particular, cardioception.

## Funding

None.

## Open science statement

This study was preregistered with an analysis plan at osf.io (https://doi.org/10.17605/OSF.IO/S6WU2). Data and analysis code are available at https://osf.io/rswve.

## Author contributions

The authors made substantial contributions to the following tasks of research: conceptualization and study design (A.P., A.L.G., C.W.); implementation (A.P., A.R., C.W.) acquisition of subjects (A.R., F.K.), data collection (A.R., F.K.); project administration (N.S., A.P.); data curation (A.R., F.K., N.S.); data analysis and interpretation (N.S.); writing: original draft (N.S.); writing: review & editing (A.L.G., A.P., A.R., C.W., F.K., M.W.) scientific supervision (A.P., A.L.G., M.W.) All authors read and approved the final manuscript.

## Declaration of competing interest

The authors declare that they have no known competing financial interests or personal relationships that could have appeared to influence the work reported in this paper.

## Appendix Supplementary materials

Image, application 1
