# Supplementary material for: Do expectations shape interoceptive perceptions across body domains? A sham EMF study to test the predictive processing theory
Source: Int J Clin Health Psychol. 2025 Jul 18;25(3):100609. doi: 10.1016/j.ijchp.2025.100609 (PMC12301728; doi:10.1016/j.ijchp.2025.100609)
Supplement: Supplementary file 1 [file mmc1.docx]

S1 - Table

Descriptive Statistics for Hit Rates, False Alarm Rates, Sensitivity d’, and Response Bias c in All Four Trial Types of the SSDT

|  | Control group  Mdn (IQR) | | Sham Wi-Fi group  Mdn (IQR) | |
| --- | --- | --- | --- | --- |
|  | LED on | LED off | LED on | LED off |
| Hit rate, % | 69.05 (45.24) | 52.38 (47.62) | 69.05 (38.10) | 54.76 (47.62) |
| FA rate, % | 7.14 (9.52) | 7.14 (9.52) | 7.14 (14.29) | 7.14 (9.52) |
| Sensitivity d’ | 1.71 (1.71) | 1.65 (1.54) | 1.76 (1.60) | 1.58 (1.08) |
| Response bias c | 0.52 (0.82) | 0.65 (0.65) | 0.48 (0.79) | 0.80 (0.78) |

FA = False Alarm, IQR = Interquartile Range, LED = light-emitting diode, Mdn = Median, SSDT = Somatosensory Signal Detection Task

S2 - Table

|  |  |  |  |  |  | 95%- Confidence Interval | | Correlation | | |
| --- | --- | --- | --- | --- | --- | --- | --- | --- | --- | --- |
| Model | Regression  Coefficient | Standard  Error | Beta | T | Sig. | Lower Bound | Upper  Bound | Zero order | Partial | Part |
| (constant) | 2.775 | 1.635 |  | 1.697 | .095 | -0.504 | 6.054 |  |  |  |
| c (SSDT) | 0.228 | 0.795 | 0.037 | 0.287 | .775 | -.1365 | 1.822 | 0.043 | 0.039 | 0.037 |
| STAI-T | 0.109 | 0.038 | 0.363 | 2.839 | .006 | 0.032 | 0.186 | 0.364 | 0.363 | 0.363 |

Regression Model predicting PHQ-15 by Response Bias (*SSDT*) and Trait Anxiety in the *EMF* Group

S3 – Table

Regression Model predicting PHQ-15 by Response Bias (*SSDT*) and Trait Anxiety in the *Control* Group

|  |  |  |  |  |  | 95%- Confidence Interval | | Correlation | | |
| --- | --- | --- | --- | --- | --- | --- | --- | --- | --- | --- |
| Model | Regression  Coefficient | Standard  Error | Beta | T | Sig. | Lower Bound | Upper  Bound | Zero order | Partial | Part |
| (constant) | 1.430 | 1.850 |  | 0.773 | .443 | -2.282 | 5.142 |  |  |  |
| c (SSDT) | 0.432 | 0.947 | 0.059 | 0.456 | .650 | -1.469 | 2.333 | 0.118 | 0.063 | 0.058 |
| STAI-T | 0.131 | 0.046 | 0.372 | 2.871 | .006 | 0.039 | 0.223 | 0.381 | 0.370 | 0.367 |

S4 – Table

Regression Model predicting PHQ-15 by Response Bias (*cvSDT*) and Trait Anxiety in the *EMF* Group

|  |  |  |  |  |  | 95%- Confidence Interval | | Correlation | | |
| --- | --- | --- | --- | --- | --- | --- | --- | --- | --- | --- |
| Model | Regression  Coefficient | Standard  Error | Beta | T | Sig. | Lower Bound | Upper  Bound | Zero order | Partial | Part |
| (constant) | 2.806 | 1.396 |  | 2.011 | .050 | -0.003 | 5.615 |  |  |  |
| c (cvSDT) | -2.324 | 0.906 | -0.325 | -2.565 | .014 | -4.149 | -0.500 | -0.297 | -0.354 | -0.324 |
| STAI-T | 0.112 | 0.034 | 0.420 | 3.310 | .002 | 0.044 | 0.181 | 0.398 | 0.439 | 0.419 |

S5 – Table

Regression Model predicting PHQ-15 by Response Bias (*cvSDT*) and Trait Anxiety in the *Control* Group

|  |  |  |  |  |  | 95%- Confidence Interval | | Correlation | | |
| --- | --- | --- | --- | --- | --- | --- | --- | --- | --- | --- |
| Model | Regression  Coefficient | Standard  Error | Beta | T | Sig. | Lower Bound | Upper  Bound | Zero order | Partial | Part |
| (constant) | 1.056 | 1.797 |  | 0.588 | .559 | -2.556 | 4.668 |  |  |  |
| c (cvSDT) | 0.507 | 1.100 | 0.060 | 0.461 | .647 | -1.704 | 2.717 | 0.076 | 0.066 | 0.060 |
| STAI-T | 0.144 | 0.045 | 0.414 | 3.191 | .002 | 0.053 | 0.234 | 0.416 | 0.415 | 0.414 |

S6 – Table

Regression Model predicting CES by Response Bias (*SSDT*) and State Anxiety in the *EMF* Group

|  |  |  |  |  |  | 95%- Confidence Interval | | Correlation | | |
| --- | --- | --- | --- | --- | --- | --- | --- | --- | --- | --- |
| Model | Regression  Coefficient | Standard  Error | Beta | T | Sig. | Lower Bound | Upper  Bound | Zero order | Partial | Part |
| (constant) | 6.315 | 2.732 |  | 2.312 | .025 | 0.836 | 11.794 |  |  |  |
| c (SSDT) | -2.681 | 1.149 | -0.262 | -2.332 | .024 | -4.986 | -0.375 | -0.353 | -0.305 | -0.257 |
| STAI-T | 0.272 | 0.063 | 0.488 | 4.344 | .000 | 0.147 | 0.398 | 0.537 | 0.512 | 0.479 |

S7 – Table

Regression Model predicting CES by Response Bias (*SSDT*) and State Anxiety in the *Control* Group

|  |  |  |  |  |  | 95%- Confidence Interval | | Correlation | | |
| --- | --- | --- | --- | --- | --- | --- | --- | --- | --- | --- |
| Model | Regression  Coefficient | Standard  Error | Beta | T | Sig. | Lower Bound | Upper  Bound | Zero order | Partial | Part |
| (constant) | 11.248 | 2.410 |  | 4.667 | .000 | 6.407 | 16.089 |  |  |  |
| c (SSDT) | -0.143 | 1.060 | -0.019 | -0.135 | .893 | -2.273 | 1.987 | 0.005 | -0.019 | -0.019 |
| STAI-T | 0.072 | 0.067 | 0.150 | 1.060 | .294 | -0.064 | 0.207 | 0.147 | 0.148 | 0.148 |

S8 – Table

Regression Model predicting CES by Response Bias (*cvSDT*) and State Anxiety in the *EMF* Group

|  |  |  |  |  |  | 95%- Confidence Interval | | Correlation | | |
| --- | --- | --- | --- | --- | --- | --- | --- | --- | --- | --- |
| Model | Regression  Coefficient | Standard  Error | Beta | T | Sig. | Lower Bound | Upper  Bound | Zero order | Partial | Part |
| (constant) | 5.012 | 2.540 |  | 1.973 | .055 | -0.102 | 10.125 |  |  |  |
| c (cvSDT) | -2.305 | 1.350 | -0.209 | -1.707 | .094 | -5.022 | 0.412 | -0.296 | -0.244 | -0.206 |
| STAI-T | 0.257 | 0.063 | 0.502 | 4.100 | .000 | 0.131 | 0.383 | 0.538 | 0.517 | 0.494 |

S9 – Table

Regression Model predicting CES by Response Bias (*cvSDT*) and State Anxiety in the *Control* Group

|  |  |  |  |  |  | 95%- Confidence Interval | | Correlation | | |
| --- | --- | --- | --- | --- | --- | --- | --- | --- | --- | --- |
| Model | Regression  Coefficient | Standard  Error | Beta | T | Sig. | Lower Bound | Upper  Bound | Zero order | Partial | Part |
| (constant) | 11.199 | 2.381 |  | 4.704 | .000 | 6.415 | 15.983 |  |  |  |
| c (cvSDT) | 0.357 | 1.270 | 0.040 | 0.282 | .779 | -2.194 | 2.909 | 0.062 | 0.040 | 0.040 |
| STAI-T | 0.068 | 0.066 | 0.146 | 1.024 | .311 | -0.066 | 0.202 | 0.152 | 0.145 | 0.145 |
